# Exhaled breath particles as a diagnostic tool for bronchiolitis obliterans syndrome in lung transplant recipients: a longitudinal study

**DOI:** 10.3389/frtra.2025.1516728

**Published:** 2025-05-23

**Authors:** Runchuan Gu, Embla Bodén, Sandra Lindstedt, Franziska Olm

**Affiliations:** ^1^ Department of Clinical Sciences, Lund University, Lund, Sweden; ^2^ Wallenberg Centre for Molecular Medicine, Lund University, Lund, Sweden; ^3^ Lund Stem Cell Centre, Lund University, Lund, Sweden; ^4^ Department of Cardiothoracic Surgery and Transplantation, Skåne University Hospital, Lund, Sweden

**Keywords:** lung transplantation, exhaled breath particles, diagnostic marker, chronic rejection, chronic lung allograft dysfunction, bronchiolitis obliterans syndrome, particle flow rate

## Abstract

**Background:**

Long-term survival after lung transplantation is significantly shorter compared with other solid organ transplantations. Chronic lung allograft dysfunction (CLAD), including bronchiolitis obliterans syndrome (BOS), remains the major barrier to survival. CLAD is diagnosed according to ISHLT's guidelines: a 20% drop in FEV_1_ using spirometry for CLAD grade 1. Given the difficulties of confounders using spirometry, other methods for precise diagnostics are being explored. Exhaled breath particles (EBP) measured as particle flow rate (PFR) from the airways have been explored as a potential method to diagnose lung injury in preclinical and clinical settings of acute respiratory distress syndrome (ARDS) and primary graft dysfunction (PGD). In fact, PFR has been shown to indicate early signs of lung injury in both ARDS and PGD settings. In the present study, we explored whether PFR could be used as a marker for BOS.

**Methods:**

Lung transplant patients with different BOS grades were included. All patients were in stable condition without ongoing infections and >2 years posttransplantation. PFR (in particles per liter) was measured using a Particles in Exhaled Air (PExA) 2.0 device (PExA, Gothenburg, Sweden), containing an optical particle counter, at the start of the study and then 1 year out, in total two time points (0 and 1 year). Particles in the diameter range of 0.41–4.55 µm were measured.

**Results:**

At both the start of the study and 1 year out, patients with BOS grade 0 had significantly higher PFR than patients with BOS grades 2–3. During the study period, patients who progressed in their BOS grade all expressed lower PFR as they progressed in BOS grade, while patients who remained stable in BOS grade did not. The particle distribution between the different BOS grades had a similar pattern; however, it significantly decreased PFR with severity in the BOS grade.

**Conclusions:**

EBP expressed as PFR could be used to distinguish severity in BOS grade and could be used to follow the progression of BOS over time. PFR could be used as a new diagnostic tool for BOS and to follow the development of lung function over time.

## Introduction

Lung transplantation (LTx) is currently the only treatment option for end-stage pulmonary disease. Despite improvement during the last years, the 1- and 5-year survival rates following LTx are still very low compared with other solid organ transplantations (1). The limited survival is primarily due to the occurrence of primary graft dysfunction (PGD), the main limitation of early survival, and chronic lung allograft dysfunction (CLAD), the main limitation for long-term survival (2–5). The development of CLAD is characterized by a progressive and non-infectious decline in lung function and affects up to 50% of all LTx recipients within the first 5 years after transplantation and 75% within 10 years (1, 6, 7). There are two major phenotypes of CLAD: bronchiolitis obliterans syndrome (BOS) and restrictive allograft syndrome (8). Although the prognosis for patients diagnosed with CLAD is generally poor, it has been shown that early diagnosis helps optimize the available therapies and prolong patient survival (9). As of today, the diagnosis mainly relies on spirometry, high-resolution computed tomography, and transbronchial lung biopsies (9–11). However, these methods are somewhat limited by low sensitivity and specificity. Due to this, a considerable and permanent decrease in lung function may already have developed by the time a CLAD diagnosis is set (12–14). There is therefore a clear need to improve and optimize the diagnostic methods for CLAD.

To date, several different techniques have been explored for non-invasively monitoring the status of the lung. The technique of sampling exhaled breath particles (EBP) has emerged as an attractive alternative to conventional techniques because it is non-invasive and allows repeated sampling with ease and no risk for the patient (14, 15). Exhaled breath particles are formed in the distal airways and have been shown to reflect the overall chemical composition of the respiratory tract lining fluid (15–20). We have previously demonstrated that EBP expressed as particle flow rate (PFR) from the airways (PFR), as an on-site and direct measurement of EBP, can be used to diagnose both PGD in lung transplant patients and acute respiratory distress syndrome (ARDS) in COVID-19 patients, as well as in pre-clinical settings in porcine models (19, 21–27). However, PFR has not yet been evaluated for BOS. In the present prospective observational study, we hypothesized that there is an association between PFR and the onset of BOS as well as BOS severity.

## Materials and methods

### Ethical considerations

The study was performed in accordance with the Declaration of Helsinki and was approved by the Swedish Ethical Board (Dnr 2017/396). All patients gave written informed consent before participating in the study.

### Patients and samples

Patients were included based on the following inclusion and exclusion criteria:

Inclusion criteria:

• Patients over the age of 18 years
• Patients who underwent double lung transplantation
• Double lung transplantation patients at least 2 years posttransplant
• Patients with BOS grades 0–3

Exclusion criteria:

• Patients diagnosed with active or invasive infection (patients with colonization were not excluded)
• Patients with other forms of CLAD than BOS, e.g., restrictive allograft syndrome
• Lack of informed consent

Eligible patients (*n* = 40) that were clinically diagnosed with CLAD were phenotyped as BOS and assigned to BOS grades based on pulmonary function tests, chest imaging, and transbronchial biopsies according to the International Society for Heart and Lung Transplantation/American Thoracic Society/European Respiratory Society (ISHLT/ATS/ERS) clinical practice guidelines statement from 2014 (9) which were followed at time of diagnosis for all patients. Accordingly, BOS grades were determined according to the decline of forced expiratory volume in 1 s (FEV_1_) relative to the patient's baseline FEV_1_. All patients included in the study exhibited an obstructive phenotype and were further diagnosed with BOS. For clarity, BOS grading (Stages 0–3) was used throughout the analysis to classify disease severity which was defined as follows:

BOS grade 0: FEV_1_ decline of <10%–19%
BOS grade 1: FEV_1_ decline of 20%–34%
BOS grade 2: FEV_1_ decline of 35%–49%
BOS grade 3: FEV_1_ decline of ≥50%

In comparison, the recent ISHLT guidelines from 2019 (28) define the CLAD grades as follows:

CLAD 0: current FEV_1_ > 80 FEV_1_ baseline
CLAD 1: current FEV_1_ > 65%‒80% FEV_1_ baseline
CLAD 2: current FEV_1_ > 50%‒65% FEV_1_ baseline
CLAD 3: current FEV_1_ > 35%‒50% FEV_1_ baseline
CLAD 4: current FEV_1_ ≤ 35% FEV_1_ baseline

Spirometry measures were combined with evaluation including imaging and bronchoscopy to identify and rule out other specific causes (9). To minimize potential confounding from early postoperative complications, only patients who were at least 2 years post-lung transplantation were included. Patients receiving inhalation therapy were sampled at least four hours post-inhalation to minimize any potential interference with PFR measurements. Among the 40 patients, 24 were BOS grade 0, 7 were grade 1, 5 were grade 2, and 4 were grade 3. Samples were obtained at baseline following double lung transplantation, and of those 40 patients, 32 were sampled again after 1 year. Three patients were excluded due to re-transplantation secondary to graft failure, and five patients died before the 1-year follow-up. Six patients progressed in their BOS grade from the first measurement at baseline to the 1-year follow-up. Patients whose BOS grades were higher at the 1-year follow-up than at baseline were regarded as having progressive BOS, and patients whose BOS grades remained unchanged at the 1-year follow-up compared with baseline were regarded as having stable BOS. Patients with BOS grade 0 at baseline, who did not experience progression over the 1-year follow-up period, served as internal controls for time-locked comparisons. As per ISHLT guidelines (9, 28), BOS grade 0 patients are considered BOS-free at the time of assessment. These patients served as a reference group for assessing changes in PFR over time.

### Measurements of particle flow rate from the airways

The measurement of PFR was performed using the Particles in Exhaled Air (PExA) device (PExA, Gothenburg, Sweden), in which the patient breathes into the device containing a two-stage inertial impactor and an optical particle counter as previously described in detail (21). The method requires a standardized breathing maneuver, as previously described (15). The patient is required to exhale a total of 60 L of air to complete the process. However, in cases of impaired lung function, the procedure is terminated after 30 min, regardless of whether the 60 L target has been reached. The particles were analyzed and expressed as the number of particles per volume and relative counts per particle size. The measured PFR and further collected EBP onto a membrane were stratified into eight different size bins based on their inertia, ranging in size from 0.41 µm to 4.55 µm in diameter. Particle sizes 1–8 corresponded to the EBP from the smallest to the largest sizes, with particle size 1 being the smallest and particle size 8 being the largest. Data regarding the PFR, defined as the number of particles per liter of exhaled air, was also acquired. Samples were collected at baseline and again at 1-year follow-up.

### Statistical analysis

All statistical analyses were conducted using GraphPad Prism version 10.3.1. Normally distributed data are presented as the mean ± standard deviation, whereas non-parametric data are presented as the median with interquartile range (IQR). Student's *t*-test (for normally distributed data), Mann–Whitney *U* test, Wilcoxon signed-rank test, Kruskal–Wallis H test, and Dunn's test (for non-parametric data) were applied to the data to evaluate statistical differences between subgroups. Statistical significance was defined as *p* < 0.001 (***), *p* < 0.01 (**), *p* < 0.05 (*), and *p* > 0.05 (not significant).

## Results

### Descriptive results

**Figure 1:**
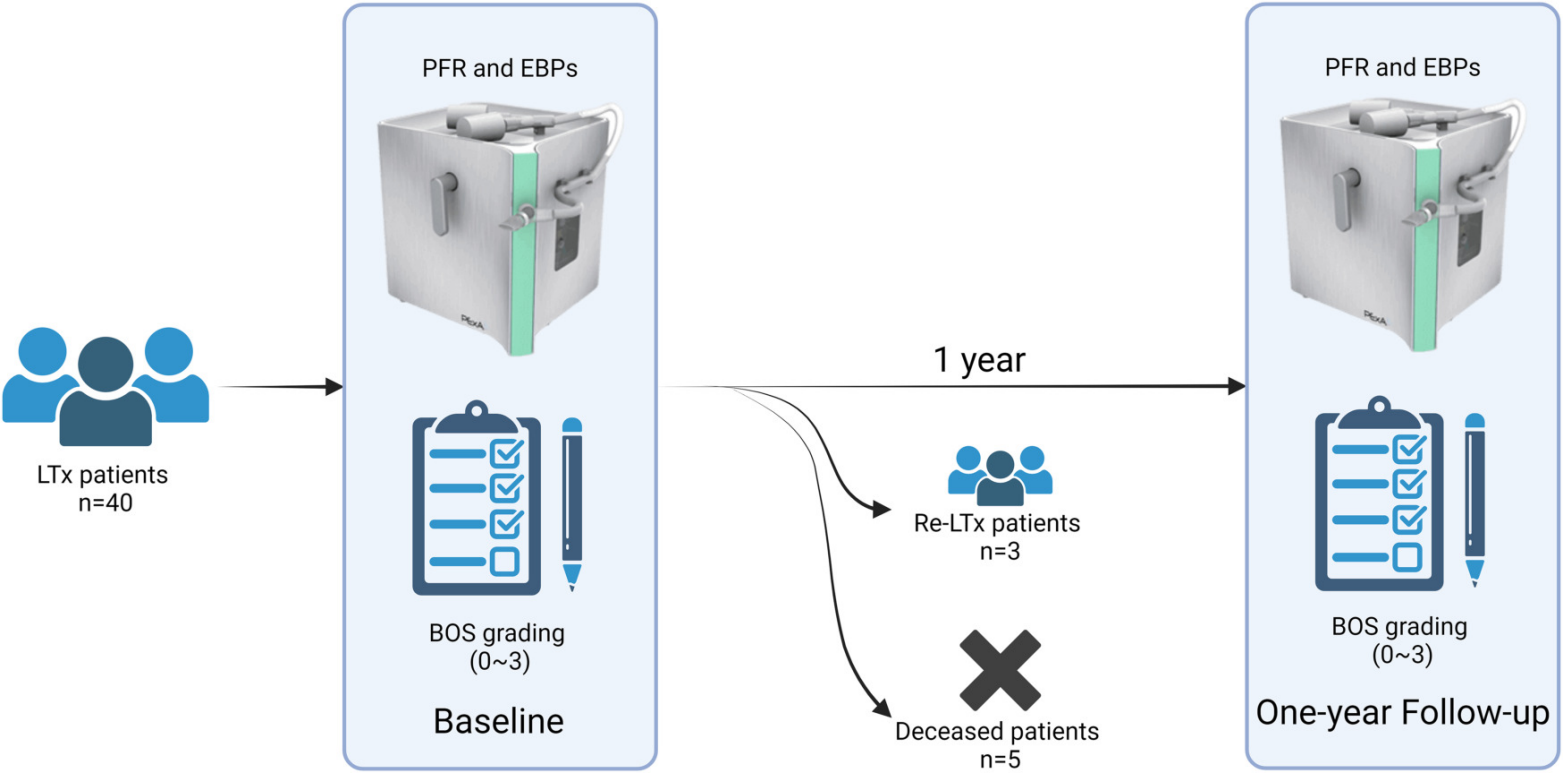
Study overview. The current study includes a total of 40 lung transplanted (LTx) recipients undergoing double lung transplantation with varying grades of BOS. All patients were sampled for EBP and PFR at the baseline timepoint, and 32 patients were sampled again 1 year later at the 1-year follow-up. Eight patients were unable to complete the 1-year follow-up measurement due to five cases of death (deceased patients) and three cases of re-transplantation secondary to graft failure (Re-LTx patients). BOS, bronchiolitis obliterans syndrome; PFR, particle flow rate; EBP, exhaled breath particles; Re-LTx, re-transplantation.

Of the 40 patients included at baseline, 28 (58%) were female, and the median age in the cohort was 55 years (IQR: 21–73). All patients underwent a double lung transplantation. The major indications for LTx included chronic obstructive pulmonary disease (COPD) (*n* = 5), cystic fibrosis (*n* = 11), alpha-1 antitrypsin deficiency (*n* = 9), pulmonary fibrosis (*n* = 7), pulmonary hypertension (*n* = 5), and other (*n* = 3). The category other includes bronchiectasis, sarcoidosis, and graft-vs.-host disease. A total of 24 patients had BOS grade 0 at baseline, seven had BOS grade 1, 5 had BOS grade 2, and 4 had BOS grade 3. Thirty-two of the included patients were sampled again at the 1-year follow-up. Patients who underwent re-transplantation or patients who died before the 1-year follow-up were not sampled more than once (Figure 1). Patient characteristics are summarized in Table 1. A total of six patients experienced progression of BOS grade between baseline and 1-year follow-up.

**Table 1:** Patient characteristics (*n* = 40). Eligible BOS patients included those in stable condition, without ongoing infection.

Variable	Value
Sex; female	23 (57.5%)
Age at LTx, years	55 (21–73)
Diagnosis	
COPD	5 (12.5%)
Cystic fibrosis	11 (27.5%)
A1ATD	9 (22.5%)
PF	7 (17.5%)
PH	5 (12.5%)
Other	3 (7.5%)
BOS grade 0	24 (60%)
BOS grade 1	7 (17.5%)
BOS grade 2	5 (12.5%)
BOS grade 3	4 (10%)
FEV_1_ at baseline timepoint	
BOS grade 0	2.5 ± 0.7
BOS grade 1	2.3 ± 0.5
BOS grades 2–3	1.3 ± 0.6
TLC at baseline timepoint	
BOS grade 0	5.7 ± 1.2
BOS grade 1	5.4 ± 0.9
BOS grades 2–3	5.8 ± 2.0
FEV_1_ at 1-year follow-up	
BOS grade 0	2.5 ± 0.7
BOS grade 1	2.0 ± 0.6
BOS grades 2–3	1.4 ± 0.4
TLC at 1-year follow-up	
BOS grade 0	5.7 ± 1.4
BOS grade 1	4.9 ± 1.0
BOS grades 2–3	5.1 ± 1.0

Numbers are expressed as the mean ± SD when parametric and median (range) when values are non-parametric or numerical (%). LTx, lung transplantation; COPD, chronic obstructive pulmonary disease; A1ATD, alpha-1 antitrypsin deficiency; PF, pulmonary fibrosis; PH, pulmonary hypertension; Other includes bronchiectasis, sarcoidosis, and graft-vs.-host disease; BOS, bronchiolitis obliterans syndrome; FEV_1_, forced expiratory volume in 1 s; TLC, total lung capacity in liters (L).

### Decreasing PFR is associated with increasing severity of BOS and BOS progression

**Figure 2:**
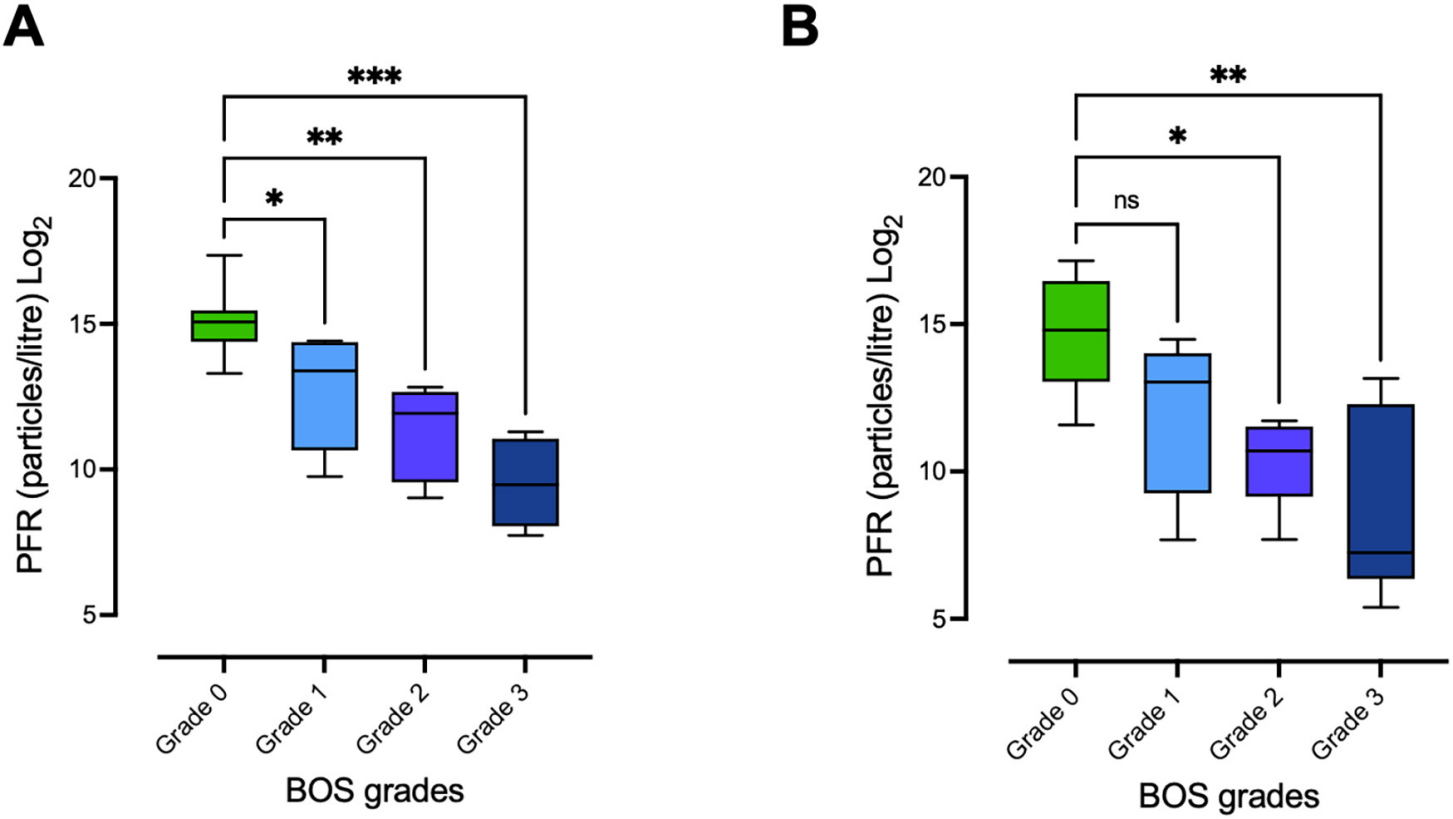
Differing PFR between LTx recipients with different BOS grades. PFR was measured at baseline and at the 1-year follow-up in lung transplant (LTx) recipients with varying grades of BOS. **(A)** PFR at baseline for patients with BOS grades 0–3. PFR in patients with more severe grades of BOS was significantly lower compared with patients with BOS grade 0. **(B)** PFR at the time of 1-year follow-up, with similar results: patients with severe BOS grades 2–3 continued to exhibit significantly lower PFR compared with those with BOS grade 0. Statistical significance was tested using the Kruskal–Wallis H test for overall comparisons among all four groups and Dunn's test for pairwise comparisons. Statistical significance is defined as *p* < 0.001 (***), *p* < 0.01 (**), *p* < 0.05 (*), and *p* > 0.05 (ns). PFR, particle flow rate; BOS, bronchiolitis obliterans syndrome.

To deepen the understanding of how PFR and EBP may be used in the setting of BOS, we first explored whether the PFR could be utilized to detect different stages of BOS. When comparing the baseline PFR values between LTx recipients with BOS grade 0 and patients with BOS grades 1–3, significant decreases in PFR could be seen in all subgroups of patients with a BOS diagnosis (BOS grades 1, 2, and 3) compared with patients with BOS grade 0 [median PFR BOS grade 0 = 34,417 (IQR: 21,457–44,991); median PFR BOS grade 1 = 10,682 (IQR: 1,615–21,190), *p* = 0.041; median PFR BOS grade 2 = 3,904 (IQR: 752–6,489), *p* = 0.003; median PFR BOS grade 3 = 715 (264.8–2,135), *p* = 0.001] (Figure 2A; Supplementary Table S1). These results remained mostly consistent at the one-year follow-up, with the PFR values of patients with BOS grades 2 and 3 being significantly lower compared with those of recipients with BOS grade 0 [median PFR BOS grade 0 = 28,512 (IQR: 8,443–90,484); median PFR BOS grade 1 = 8,335 (IQR: 609.5–16,430), *p* = 0.459; median PFR BOS grade 2 = 1,659 (IQR: 566.5–2,944), *p* = 0.048; median PFR BOS grade 3 = 152 (IQR: 82–4,995), *p* = 0.005] (Figure 2B; Supplementary Table S1).

**Figure 3:**
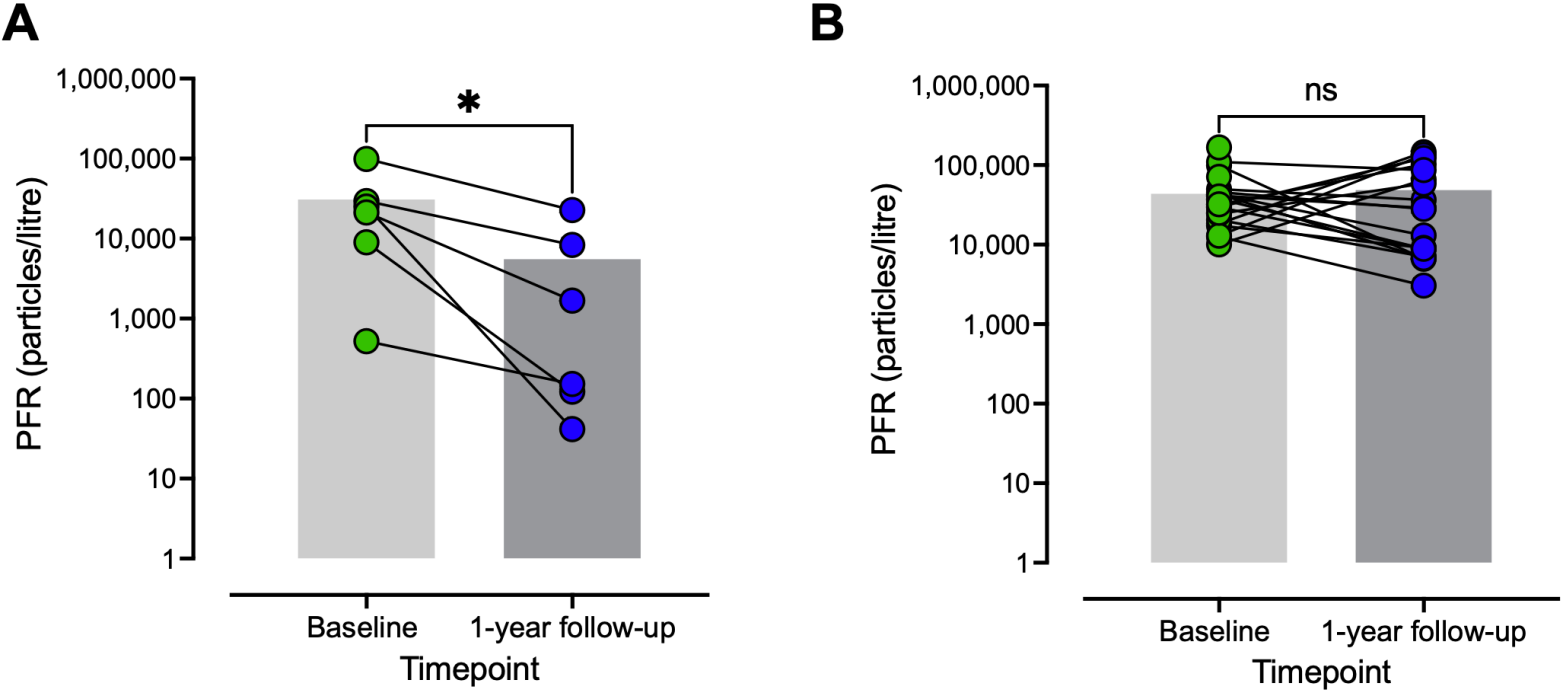
Decreasing PFR is associated with progressive BOS. **(A)** Change in PFR with progressive BOS from baseline to the 1-year follow-up. **(B)** Change in PFR for patients with stable BOS between baseline and the 1-year follow-up. Patients whose BOS grades were higher at the 1-year follow-up than at baseline were regarded as having progressive BOS, and patients whose BOS grades remained unchanged at the 1-year follow-up compared with baseline were regarded as having stable BOS. PFR was significantly reduced in patients with progressive BOS at the 1-year follow-up compared with baseline, whereas no such reduction was observed in patients with stable disease. Statistical significance was tested using the Wilcoxon test. Statistical significance is defined as *p* < 0.001 (***), *p* < 0.01 (**), *p* < 0.05 (*), and *p* > 0.05 (ns). PFR, particle flow rate; BOS, bronchiolitis obliterans syndrome; ns, not significant.

We further investigated whether lower PFR values were associated with progressive BOS, and we found significantly lower PFR values at 1-year follow-up in LTx recipients with progression of BOS compared with baseline values [median PFR at baseline = 23,199 (IQR: 6,872–46,892); median PFR at 1-year follow-up = 912.5 (101.9–11,948), *p* = 0.031] (Figure 3A; Supplementary Table S2). On the other hand, patients with stable disease showed no significant decrease in PFR between the two timepoints [median PFR at baseline = 30,818 (IQR: 18,204–43,730); median PFR at one-year follow-up = 28,512 (IQR: 8,443–90,484), *p* = 0.932] (Figure 3B; Supplementary Table S2).

### Similar particle size distribution patterns between different BOS grades

Particle size distribution patterns of collected EBP samples were also analyzed; however, this showed no striking differences in either shape or size of the curves, regardless of BOS grades. This observation held true for both baseline and 1-year follow-up measurements. The total number of collected particles in each of the eight size bins was also compared between patients with different grades of BOS. At baseline, an increase in accumulated particles was observed in the three smallest size bins for patients with higher BOS grades, and furthermore, a decrease in accumulated particles was noted in the medium and larger size bins for patients with higher BOS grades, both at baseline and at the 1-year follow-up (Supplementary Table S3).

**Figure 4:**
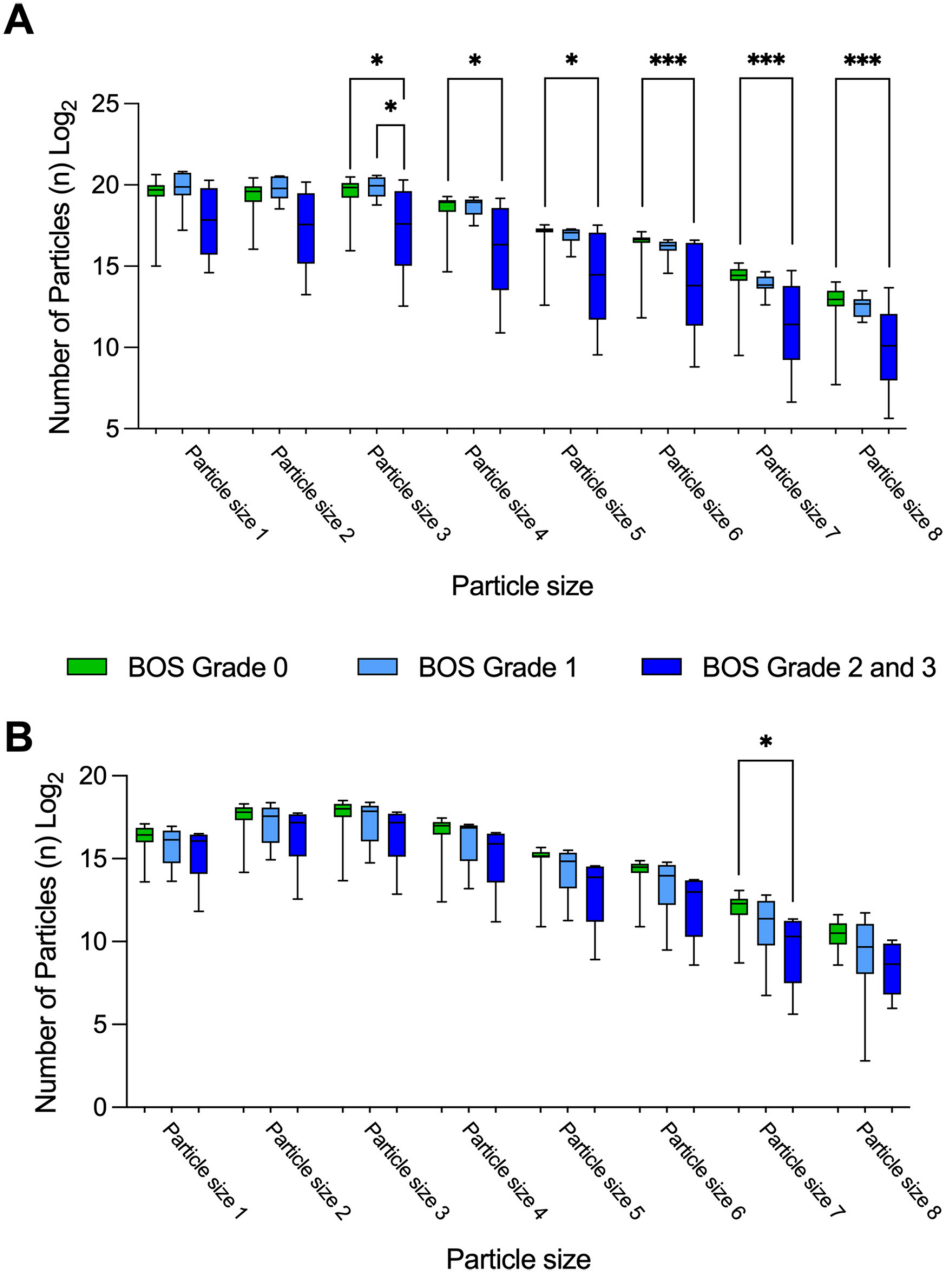
Particle size distribution patterns depend on BOS grade. Particle size distribution patterns for EBP were analyzed at baseline and at the 1-year follow-up, categorized by BOS grade. **(A)** Particle size distribution at baseline, showing significant differences in the quantities of larger EBP sizes between patients with BOS grade 0 and those with BOS grades 2–3. **(B)** Particle size distribution at the 1-year follow-up, demonstrating similar trends to those observed at baseline. Statistical significance was tested using the Kruskal–Wallis H test and Dunn's test. Statistical significance is defined as *p* < 0.001 (***), *p* < 0.01 (**), *p* < 0.05 (*), and *p* > 0.05 (ns). *N*, number of; EBP, exhaled breath particle; BOS, bronchiolitis obliterans syndrome.

During the collection of exhaled particles, the PExA device was used to count and collect EBPs. The device's impactor separated the particles by size during counting, and these size variations were analyzed (Figure 4). At baseline, significant differences in particle sizes, particularly in the larger size bins, were observed. At the 1-year follow-up, differences in particle sizes were noted but were not as pronounced as at baseline. It is possible that some patients with progressive BOS had not yet been diagnosed at that point. Moreover, particles of larger sizes can fracture into smaller components due to the mechanical impact while traveling into the larger respiratory airways, further clouding the interpretation of these patterns (22, 23, 28).

## Discussion

Particle flow rate (PFR) from the airways has emerged as a promising non-invasive alternative to conventional diagnostic methods, offering the advantage of repeated, risk-free sampling. Our team has previously demonstrated that PFR can effectively diagnose PGD in lung transplant recipients and acute respiratory distress syndrome (ARDS) in COVID-19 patients (19–27). However, its application in diagnosing BOS has remained unexplored. In this prospective observational study, we demonstrate that PFR can reliably diagnose BOS and distinguish between varying degrees of disease severity.

Given that BOS leads to lung injury and a decrease in lung function, we hypothesized that PFR could serve as a tool to assess and monitor lung function in patients across different stages of BOS. To date, no study has investigated the use of PFR in this context. In the present study, we observed a correlation between PFR values and BOS severity, revealing a clear association: higher BOS grades corresponded to lower PFR values. This relationship was also evident when comparing patients with progressive BOS to those with stable disease, where lower PFR values were linked to more severe rejection.

We observed a clear distinction in PFR between patients without BOS and those with BOS grades 1–3. Additionally, it was possible to differentiate between the various BOS grades using PFR. The consistency of measurements at both baseline and 1-year follow-up further supports PFR as a potentially robust marker for lung allograft rejection.

We also evaluated PFR in patients who experienced BOS progression between baseline and 1-year follow-up, according to ISHLT guidelines (9), and compared them to those with stable respiratory function during the study period. All patients with BOS progression demonstrated significantly lower PFR levels, while patients with stable BOS grades showed no such reduction, reinforcing the potential of PFR as a potential reliable diagnostic tool for BOS.

We propose that PFR potentially offers a safe and informative method for both early detection and ongoing monitoring of BOS progression in lung transplant recipients. The absence of significant PFR reduction between BOS grades 0 and 1 at 1-year follow-up may be partially explained by the variability in PFR values among patients with BOS grade 0, potentially indicating undiagnosed cases of BOS.

We have previously shown that acute inflammatory states of the lung result in increased PFR (21, 24). The increase in PFR demonstrated in earlier publications in settings of ARDS and PGD is logical, because both ARDS and PGD are associated with massive inflammatory responses within the alveoli. During this process, especially the distal airways are infiltrated by a host of inflammatory cells, and a cytokine storm can be seen, as well as the development of edema, both within the interstitial space and within the respiratory tracts themselves. The increase in fluids within the lungs increases the amount of respiratory tract lining fluid from which EBP is generated, and this is believed to be the origin of the increased amount of EBP as well as elevated PFR among patients with PGD or ARDS (21, 22, 24, 26). BOS is a chronic change in the lung, with the pathophysiology being obstructive by nature and the diagnosis being characterized by pathological changes to the bronchioles with obliteration and fibrosis of the pulmonary tissue and loss of alveolar attachments, leading to mechanically reduced potential for exhalation of EBP as the peripheral airways are the main source for these particles (29). The mechanical blockage of the air flow through the respiratory tract may explain the significant drop in PFR among patients with BOS. This is supported by earlier publications, stating that patients with other forms of obstructive pulmonary disease, such as asthma, show similar phenomena with a decrease in PFR (30). While the reduction in PFR is likely related to fibrotic airway obstruction in BOS, other mechanisms such as epithelial injury, mucus accumulation, and altered surfactant dynamics may also play a role. Future studies incorporating compositional analysis of exhaled breath particles may provide deeper mechanistic insights into BOS pathophysiology.

While BOS is diagnosed according to ISHLT guidelines (9), challenges remain in distinguishing BOS from infections and establishing reliable baselines. Given these limitations, there is growing interest in alternative diagnostic tools, including cf-DNA, dd-DNA, and exosomes. Comparable to spirometry, PFR must be measured longitudinally to serve as a practical clinical tool, requiring further studies to establish threshold values for disease progression, particularly in high-risk patients where complementary spirometry may enhance early detection. While PFR also requires a breathing maneuver, it relies on a controlled effort rather than a maximal forced expiration, which may improve feasibility in certain patient populations and enable earlier detection of small airway changes before significant FEV₁ decline occurs.

Future studies may benefit from integrating PFR with high-resolution CT imaging to explore the relationship between global airway function and regional anatomical changes, such as small airway dimensions or air trapping. Such an approach would require advanced imaging protocols and computational tools, offering a promising avenue for a more detailed understanding of BOS pathology. This study represents the first exploratory investigation into the feasibility of EBP expressed as PFR as a diagnostic marker, highlighting the potential and the need for further research and validation to establish its clinical utility. The use of PFR measurements, guided by trained personnel, has proven feasible in clinical research settings. With the device already being employed in several research centers, it holds promise for future broader clinical adoption with appropriate training and standardization.

## Limitation

The current study represents the first exploratory investigation into the feasibility of using EBP as a potential diagnostic tool for BOS and its progression. Patients with BOS grade 0 at baseline who did not experience progression over the 1-year follow-up served as internal controls for time-locked comparisons, and future studies could further strengthen these findings by including external control groups for more robust validation. Furthermore, to establish EBP as a reliable early diagnostic biomarker, future studies with more frequent longitudinal measurements will be necessary. While it is too early to determine whether EBP could outperform existing methods, future studies integrating comparative analyses, such as ROC and AUC curves, will be essential to further evaluate its clinical utility.

To assess the potential of EBP in reflecting BOS status, we conducted two separate PFR measurements. However, evaluating the treatment response, particularly in less stable cases, would require larger cohorts with longitudinal assessments. Future studies should incorporate investigational subgroups with neutrophilic BOS, measuring PFR at multiple time points alongside azithromycin administration to better assess its responsiveness to treatment.

Future studies should explore the integration of PFR with inflammatory biomarkers, such as immune cell infiltration, antibody titers, and cytokine levels, to provide a more comprehensive assessment of its potential as a diagnostic marker. This approach could enhance the understanding of EBP's clinical utility and represents a promising direction for follow-up research.

The patients included in this study were diagnosed based on the BOS grading system (9) rather than the latest CLAD staging framework based on the 2019 ISHLT consensus guidelines (28) to ensure consistency with prior BOS-focused studies and alignment with the original dataset’s clinical classification.

## Conclusion

In summary, our findings support the potential use of PFR and EBP analysis as a non-invasive, safe, and informative approach for both the early detection and ongoing monitoring of chronic rejection in the form of BOS in lung transplant recipients. This method holds potential for improving patient outcomes by enabling earlier intervention and more precise tracking of BOS progression, ultimately contributing to better management of lung allograft dysfunction. Future studies should further explore the mechanistic links between EBP characteristics and lung pathology, as well as evaluate the broader applicability of this approach across diverse patient populations and the integration of PFR with inflammatory biomarkers.

## Data Availability

The raw data supporting the conclusions of this article will be made available by the authors, without undue reservation.
