# Supplementary material for: Exhaled breath particles as a diagnostic tool for bronchiolitis obliterans syndrome in lung transplant recipients: a longitudinal study
Source: Front Transplant. 2025 May 23;4:1516728. doi: 10.3389/frtra.2025.1516728 (PMC12141230; doi:10.3389/frtra.2025.1516728)
Supplement: Supplementary file 1 [file Table1.docx]

**Supplementary Information**

**Supplementary Tables**

**Supplementary Table 1: Differing PFR between LTx patients with different BOS grades compared to BOS grade 0**

| **BOS grade** | **Baseline PFR, median (IQR) (particles/litre exhaled air)** | **p-value** | **One-year follow-up PFR, median (IQR) (particles/litre exhaled air)** | **p-value** |
| --- | --- | --- | --- | --- |
| **BOS grade 0** | 34417 (21457 – 44991) |  | 28512 (8443 – 90484) |  |
| **BOS grade 1** | 10682 (1615 – 21190) | 0.041* | 8335 (609.5 – 16430) | 0.459 |
| **BOS grade 2** | 3904 (752 – 6489) | 0.003** | 1659 (566.5 – 2944) | 0.048* |
| **BOS grade 3** | 715 (264.8 – 2135) | 0.001** | 152 (82 – 4995) | 0.005** |

Data are expressed as median and interquartile range (IQR: Q25 – Q75). Overall statistical significance between the subgroups was tested using the Kruskal Wallis H test, and pairwise comparisons were performed using Dunn’s test. Significant differences were identified between PFR values of patients with BOS grade 0 and patients with BOS grades 2 and 3, both at the baseline and at one-year follow-up. Furthermore, a significant difference was also observed between PFR values of patients with BOS grade 0 and patients with BOS grade 1 at baseline. Statistical significance was defined as p < 0.001 (***), p < 0.01 (**), p < 0.05 (*), and p > 0.05 (ns, non-significant, not displayed here). *PFR: particle flow rate, IQR: interquartile range, BOS: bronchiolitis obliterans syndrome, LTx: lung transplantation.*

**Supplementary Table 2: Significantly lower PFR at one-year follow-up for patients with progressive BOS**

| **BOS Progression** | **Baseline PFR, median (IQR) (particles/litre exhaled air)** | **One-year Follow-up PFR, median (IQR) (particles/litre exhaled air)** | **p-value** |
| --- | --- | --- | --- |
| **Yes** | 23199 (IQR: 6872 – 46892) | 912.5 (101.9 – 11948) | 0.031* |
| **No** | 30818 (IQR: 18204 – 43730) | 28512 (IQR: 8443 – 90484) | 0.932 |

Patients whose BOS grades were higher at the one-year follow-up than baseline were regarded as having progressive BOS, and patients whose BOS grades remained unchanged at the one-year follow-up compared to baseline were regarded as being stable. Data are expressed as median and interquartile range (IQR: Q25 – Q75). Statistical significance of PFR changes between measurements at baseline and one-year follow-up was tested in patients with and without progression of BOS via Wilcoxon test. This result showed a significant decrease in PFR in patients with progression of BOS, while patients with stable disease demonstrated no such change. Statistical significance was tested using the Wilcoxon test and was defined as p < 0.001 (***), p < 0.01 (**), p < 0.05 (*), and p > 0.05 (ns, non-significant, not displayed here). *PFR: particle flow rate, IQR: interquartile range, BOS: bronchiolitis obliterans syndrome, LTx: lung transplantation.*

**Supplementary Table 3: Differences in amounts of particles in the different size bins at baseline and one-year follow-up**

**A:** Baseline measurement

| **Size bin** | **N particles, BOS grade 0, median (IQR)** | **N particles, BOS grade 1, median (IQR)** | **N particles, BOS grades 2 & 3, median (IQR)** | **p-value** |
| --- | --- | --- | --- | --- |
| **1** | 847310 (639678 – 1044785) | 962290 (670170 – 1764630) | 234930 (54020 – 918110) | 0.042* |
| **2** | 796790 (506718 – 993025) | 900580 (595180 – 1520580) | 194150 (36630 – 740170) | 0.024* |
| **3** | 937415 (607403 – 1143555) | 1013920 (638700 – 1452360) | 197910 (33250 – 801290) | 0.016* |
| **4** | 498535 (332295 – 550605) | 500310 (296810 – 560120) | 82140 (11740 – 391620) | 0.017* |
| **5** | 150585 (138448 – 163248) | 137120 (96460 – 158040) | 22860 (3350 – 136950) | 0.014* |
| **6** | 101890 (89055 – 110960) | 79030 (63330 – 93520) | 14320 (2600 – 88420) | <0.001*** |
| **7** | 22030 (17663 – 28955) | 14750 (12550 – 20980) | 2750 (600.0 – 14050) | <0.001*** |
| **8** | 7955 (5898 – 11490) | 6560 (3770 – 8050) | 1100 (250.0 – 4260) | 0.001** |

**B:** One-year follow-up measurement

| **Size bin** | **N particles, BOS grade 0, median (IQR)** | **N particles, BOS grade 1, median (IQR)** | **N particles, BOS grades 2 & 3, median (IQR)** | **p-value** |
| --- | --- | --- | --- | --- |
| **1** | 88223 (65288 – 118645) | 72267 (27261 – 106304) | 68594 (17322 – 89480) | 0.351 |
| **2** | 228661 (164741 – 282797) | 193658 (63558 – 279248) | 147224 (35883 – 208531) | 0.217 |
| **3** | 263315 (186744 – 323056) | 236623 (67689 – 301132) | 148340 (35606 – 215332) | 0.097 |
| **4** | 130827 (90038 – 152435) | 118469 (29887 – 132681) | 61266 (12112 – 93056) | 0.065 |
| **5** | 36773 (34105 – 43211) | 29290 (9529 – 42130) | 15095 (2359 – 23695) | 0.089 |
| **6** | 23108 (17982 – 26524) | 15983 (4778 – 25060) | 8160 (1246 – 13269) | 0.047* |
| **7** | 5020 (3116 – 6124) | 2663 (876.8 – 5610) | 1258 (180.8 – 2446) | 0.037* |
| **8** | 1452 (907.0 – 2191) | 819.5 (265.0 – 2143) | 399.0 (112.0 – 945.5) | 0.056 |

Data are expressed as median and interquartile range (IQR: Q25 – Q75). Overall statistical significance between the subgroups was tested using the Kruskal Wallis H test and pairwise comparisons were tested using Dunn’s test. Due to small sample sizes, BOS grades 2 and 3 were combined for the purpose of statistical analysis. Significant differences can be identified in all size bins at baseline and only in two of the larger size bins at one-year follow-up. Statistical significance was defined as p < 0.001 (***), p < 0.01 (**), p < 0.05 (*), and p > 0.05 (ns, not displayed here). *N: number of, EBP: exhaled breath particle, IQR: interquartile range, BOS: bronchiolitis obliterans syndrome, LTx: lung transplantation.*
